# The Impact of Age and BMI on the VWF/ADAMTS13 Axis and Simultaneous Thrombin and Plasmin Generation in Hospitalized COVID-19 Patients

**DOI:** 10.3389/fmed.2021.817305

**Published:** 2022-01-10

**Authors:** Kiruphagaran Thangaraju, Upendra Katneni, Imo J. Akpan, Kenichi Tanaka, Tiffany Thomas, Saini Setua, Julie A. Reisz, Francesca Cendali, Fabia Gamboni, Travis Nemkov, Stacie Kahn, Alexander Z. Wei, Jacob E. Valk, Krystalyn E. Hudson, David J. Roh, Chiara Moriconi, James C. Zimring, Angelo D'Alessandro, Steven L. Spitalnik, Richard O. Francis, Paul W. Buehler

**Affiliations:** ^1^Department of Pathology, Department of Pediatrics, Center for Blood Oxygen Transport and Hemostasis, University of Maryland, Baltimore, MD, United States; ^2^Division of Hematology/Oncology, Department of Medicine, Columbia University Irving Medical Center, New York, NY, United States; ^3^Department of Anesthesiology, University of Maryland, Baltimore, MD, United States; ^4^Department of Anesthesiology, University of Oklahoma College of Medicine, Oklahoma City, OK, United States; ^5^Department of Pathology & Cell Biology, Columbia University Vagelos College of Physicians and Surgeons, New York, NY, United States; ^6^Department of Biochemistry and Molecular Genetics, University of Colorado Denver – Anschutz Medical Campus, Aurora, CO, United States; ^7^Department of Neurology, Columbia University Vagelos College of Physicians and Surgeons, New York, NY, United States; ^8^Department of Pathology, University of Virginia, Charlottesville, VA, United States

**Keywords:** COVID-19, plasmin, thrombin, von Willebrand factor, ADAMTS13

## Abstract

Aging and obesity independently contribute toward an endothelial dysfunction that results in an imbalanced VWF to ADAMTS13 ratio. In addition, plasma thrombin and plasmin generation are elevated and reduced, respectively, with increasing age and also with increasing body mass index (BMI). The severity risk of Corona Virus Disease 2019 (COVID-19) increases in adults older than 65 and in individuals with certain pre-existing health conditions, including obesity (>30 kg/m^2^). The present cross-sectional study focused on an analysis of the VWF/ADAMTS13 axis, including measurements of von Willebrand factor (VWF) antigen (VWF:AG), VWF collagen binding activity (VWF:CBA), Factor VIII antigen, ADAMTS13 antigen, and ADAMTS13 activity, in addition to thrombin and plasmin generation potential, in a demographically diverse population of COVID-19 negative (−) (*n* = 288) and COVID-19 positive (+) (*n* = 543) patient plasmas collected at the time of hospital presentation. Data were analyzed as a whole, and then after dividing patients by age (<65 and ≥65) and independently by BMI [<18.5, 18.5–24.9, 25–29.9, >30 (kg/m^2^)]. These analyses suggest that VWF parameters (i.e., the VWF/ADAMTS13 activity ratio) and thrombin and plasmin generation differed in COVID-19 (+), as compared to COVID-19 (−) patient plasma. Further, age (≥65) more than BMI contributed to aberrant plasma indicators of endothelial coagulopathy. Based on these findings, evaluating both the VWF/ADAMTS13 axis, along with thrombin and plasmin generation, could provide insight into the extent of endothelial dysfunction as well as the plasmatic imbalance in coagulation and fibrinolysis potential, particularly for at-risk patient populations.

## Introduction

Coagulopathy is a sequela of COVID-19 that associates with the severity of disease progression (1–4). Venous thrombosis and thromboembolism, as well as arterial thrombosis were reported at a relatively higher frequency in COVID-19 patients (3, 5). Microvascular coagulation and endotheliopathy are critical pathophysiological consequences of COVID-19 immune activation that contribute to death (6, 7). Microthrombi are most often observed in lung vessels at autopsy, particularly in peripheral lung venules, arterioles, and alveolar capillaries (6, 8). In survivors of severe disease, long term exertional impairments may persist due to microvascular thrombosis and consequent lung injury (8).

A focus on endothelial dysregulation has emerged based on evidence of increased von Willebrand factor (VWF) antigen (AG) levels (i.e., Ultra-large Von Willebrand Factor (ULVWF) multimers), increased VWF collagen type I and III binding activity (9), along with mild to moderately decreased ADAMTS13 AG and ADAMTS13 activity in severely ill patients (10–13). Physiologically, VWF and ADAMTS13 play important roles in the maintenance of hemostasis in the microvasculature (14). VWF is a large multimeric glycoprotein secreted as ultra-large pro-thrombotic forms into the vascular lumen, primarily from endothelial cells and platelets. Although endothelial cells show both basal and stimulated secretion, platelets release VWF only upon activation (15). Factor VIII circulates in plasma as a complex with VWF and facilitates site-specific cleavage of ULVWF multimers under shear stress (16). The release of Factor VIII from VWF occurs in the presence of thrombin leading to a 4-fold increase in its plasma clearance. ADAMTS13 is the enzyme that regulates VWF activity by digesting shear stress elongated pro-thrombotic ULVWF multimers (17). Under pathophysiological states, such as thrombotic thrombocytopenic purpura (TTP), a severe deficiency in availability or activity of ADAMTS13 (<10%) results in accumulation of pro-thrombotic VWF multimer forms leading to the formation of microvascular platelet-rich thrombi, thrombocytopenia, secondary micro-hemorrhages, and peripheral blood schistocytes (14, 18). In addition, thrombotic microangiopathies (TMA) are caused by many different pathologies, with endothelial injury being a common denominator. Interestingly, elevated VWF levels, accompanied by increased Factor VIII levels (18) as well as mildly decreased ADAMTS13 activity (~ 50%) and normal antigen levels (~ 1 U/ml), are observed in severe COVID-19 infection (19). However, a complete loss of ADAMTS13 activity (i.e., <10%), thrombocytopenia, schistocytes are not common in COVID-19 infection. Nonetheless, COVID-19 disease progression is consistent with endothelial dysfunction and increased plasma VWF levels and VWF:AG/ADAMTS13 activity ratios are associated with COVID-19 disease severity and reported to be a predictor of morbidity and mortality (10–13).

In addition to VWF/ADAMTS13 axis dysregulation, plasma predictors of thrombosis and fibrinolysis potential, such as thrombin and plasmin generation, respectively, have not been well-defined in COVID-19 patients. Similarly to VWF/ADAMTS13 axis parameter evaluation, assays that assess thrombin and plasmin could add relevant information on coagulation risk. Thrombin is the primary mediator of fibrinogen cleavage to fibrin, and thrombin generation is a useful measure of both increased and reduced coagulation potential when measured in plasma. Conversely, fibrinolysis is mediated by the proteolytic action of plasmin, which accumulates as a result of enzymatic cleavage of plasminogen by tissue plasminogen activator. Plasmin generation offers insight into the amount of available plasmin that could participate in fibrin clot lysis. Both measurements, when evaluated simultaneously, provide information on the potential for clot formation and the impairment of clot lysis, respectively. These assessments can be made prior to the onset, or during the processes of, coagulopathy, and offer relevant insight into thrombin and plasmin function in disease diagnosis, disease severity, and drug therapy assessments.

Independent of COVID-19, VWF:AG and the VWF:AG/ADAMTS13 activity ratio increase with aging (≥65 years of age) and body mass index (BMI; >25 kg/m^2^) (20, 21). Additionally, an underlying endotheliopathy is observed with aging and increasing BMI, potentially due to accumulating co-morbidities and declining organ function (22, 23). Understanding the impact of COVID-19 on endothelial markers of coagulation and more broadly, on plasma thrombin and plasmin generation, at early disease presentation may offer better insights into anticoagulation needs and monitoring as well as assessing early disease severity.

The current observational study is unique in that we evaluated the VWF/ADAMTS13 axis, as well as a simultaneous thrombin and plasmin generation assay that informs on amounts of functional thrombin and plasmin in plasma. This study evaluated individual plasmas of two large groups of demographically diverse hospitalized patients in a large urban medical center, to overcome the limitations of previous studies of endothelial dysregulation in COVID-19, which included small numbers of patients, which were then compared to healthy individuals. In contrast, we grouped hospitalized patients based on COVID-19 (−) or COVID-19 (+) status and these groups were comprised of 288 and 543 patients, respectively. Data was further evaluated based on age (i.e., <65 or ≥ 65 years), BMI (i.e., <18.5, 18.5–24.9, 25–29.9, >30 (kg/m^2^). Finally, these parameters analyzed in the present study were evaluated in surviving and non-surviving patients within the COVID-19 (−) and COVID-19 (+) groupings. The data generated were used in correlation and association analysis with age and metabolic parameters (24).

## Patients, Materials, and Methods

### Patients and Sample Collection

#### Patients

This study was approved by the Institutional Review Board of Columbia University Irving Medical Center (CUIMC) (Protocol Number AAAT0680). Data were obtained from patients who were either admitted to the hospital or seen in the Emergency Department from April 14, 2020 through May 31, 2020 (i.e., before the identification of and routine testing for novel variants in the USA), and were evaluated for SARS-CoV-2 by RT-PCR and/or serology. COVID-19 (−) patients were identified and selected based on a negative SARS-CoV-2 RT-PCR test and/or serology testing in the ED or within the initial 72 h after admission. To our knowledge, patients included in the COVID-19 (−) had no reported history of COVID-19 infection.

#### Patient Comparisons

First, patients were divided into COVID-19 (−) (*n* = 288) and COVID-19 (+) (*n* = 543) groups based on a positive SARS-CoV-2 RT-PCR test or positive serology. VWF, ADAMTS13, Factor VIII, and thrombin and plasmin generation parameters were compared between the groups.

Second, within the COVID-19 (−) and COVID-19 (+) groups, patients were split based on age <65 or age ≥ 65. Within the COVID-19 (−) group, age-dependent splitting resulted in *n* = 156 (<65 years of age) and *n* = 132 (≥65 years of age); within the COVID-19 (+) group, there were *n* = 278 patients <65 years of age and *n* = 265 patients ≥65 years of age. Comparisons for VWF, ADAMTS13, Factor VIII, and thrombin and plasmin generation parameters were made between COVID-19 (+) and (−) patients within the <65 and in the ≥65 years of age groupings. Further, parameters were compared within the COVID-19 (−) patient group based on age <65 and ≥ 65; similar comparisons were made for COVID-19 (+) patients.

Third, within COVID-19 (−) and COVID-19 (+) groups, patients were split based on CDC guidelines into four BMI categories: <18.5, 18.5–24.9, 25–29.9, >30 (kg/m^2^). Within the COVID-19 (−) group this led to the following BMI category distribution: <18.5 (*n* = 14), 18.5–24.9 (*n* = 53), 25–29.9 (*n* = 47), >30 (*n* = 92) (kg/m^2^). The COVID-19 (+) group had the following BMI category distribution: <18.5 (*n* = 15), 18.5–24.9 (*n* = 112), 25–29.9 (*n* = 98), >30 (*n* = 253) (kg/m^2^). Further, within the COVID-19 (−) and COVID-19 (+) groups, parameters were compared across BMI categorizations. All statistical analyses and graphing of data were performed using Graphpad Prism software (version 9.2.0). Data are presented as Group median values and interquartile range [25–75 percentile]. Data between COVID-19 (−) and COVID-19 (+) groups were compared using a non-parametric Mann-Whitney U test. Comparisons across several groups within BMI categorizations were analyzed with a non-parametric One-way-ANOVA with multiple comparisons using a Kruskal-Wallis test.

#### Sample Collection and Handling

All initial blood samples were collected within 72 h of admission in sodium citrate and analyzed for routine clinical laboratory values at CUIMC and processed to platelet poor plasma for research based assays (24). To maintain continuity and quality of specimens, samples arrived at the University of Maryland Baltimore under dry ice as a single shipment. Samples were analyzed in blocks (*n* = 50) to allow for a single freeze thaw followed by evaluation of enzymatic and activity assays. Plasma samples were then aliquoted into multiple tubes containing 100–200 ul and refrozen for antigen-based assays.

### VWF, ADAMTS13 and FVIII Measurements

The antigen and activity measurement of VWF and ADAMTS13 was performed by using commercial ELISA kits. VWF:AG and collagen type III binding activity (VWF:CBA) levels were measured by using Human von Willebrand Factor ELISA Kit (ab168548, Abcam, Cambridge, UK) and TECHNOZYM® vWF:CBA ELISA Kit (5450301, Technoclone, Vienna, Austria) to measure the quantity of VWF and its binding to collagen type III (therefore, an increase in VWF binding indicates more circulating ultra-large molecular weight multimers), respectively. ADAMTS13 antigen and activity levels were measured by using Human ADAMTS13 ELISA Kit (ab234559, Abcam) and TECHNOZYM® ADAMTS13 Activity ELISA (5450701, Technoclone), respectively. FVIII antigen levels were measured by using Human Factor VIII total antigen assay ELISA kit (HFVIIIKT-TOT, Molecular Innovations, Novi, MI, USA). All assays were performed following manufacturer's recommendations with additional dilution of plasma samples as required.

### Simultaneous Thrombin and Plasmin Generation Assay (STPGA)

Simultaneous measurement of thrombin and plasmin generation potential of plasma samples were performed with modifications to previous methods (25, 26). Briefly, plasma samples were mixed with 512 μM of either thrombin specific substrate, Z-Gly-Gly-Arg-AMC (Bachem, Bubendorf, Switzerland) or plasmin specific substrate, Boc-Glu-Lys-Lys-AMC (Bachem) and 16 nM of thrombomodulin (PeproTech, Rocky Hill, NJ, USA) similar to a previous method designed to measure thrombin and plasmin in parallel (26).

The reaction was initiated by adding an activator solution that yielded a final concentration of 1 pM tissue factor (Diagnostica Stago, Parsippany, NJ, USA), 0.7 μg/mL of tissue plasminogen activator (Sigma-Aldrich, St. Louis, MO, USA) and 16 mM CaCl_2_. Sample wells were supplemented with buffer (150 mM NaCl, 20 mM HEPES and pH 7.5) and AMC fluorophore instead of activator solution for background and calibrator measurements respectively. Calculation of thrombin and plasmin concentration was performed as described previously (25).

### Clinical Laboratory Data

Laboratory tests were performed based on clinical necessity and not as directed by this study; the resulting values were obtained by request from the patients' charts. Therefore, not all patients had all of the tests ordered. As part of routine care, hemostasis was evaluated on STAR Evolution and STAR Max analyzers (Diagnostica Stago, Parsippany, NJ), hematology testing by Sysmex XN900 (Lincolnshire, IL), and chemistry testing by Roche Cobas c502 (Indianapolis, IN). Laboratory values, including antithrombin (AT), prothrombin time (PT)/international normalized ratio (INR), activated partial thromboplastin time (aPTT), fibrinogen, D-dimer, white blood cell count (WBC), absolute neutrophil count (ANC), absolute lymphocyte count (ALC), absolute monocyte count (AMC), hemoglobin, red blood cell count (RBC), RBC distribution width (RDW), reticulocyte count, platelet count, IL-6, lactate dehydrogenase (LDH), lactic acid, procalcitonin, troponin, blood urea nitrogen (BUN), creatinine, glucose, bilirubin (total, direct, and indirect), aspartate amino transferase (27), alanine amino transferase (ALT), albumin, total protein, ferritin, C-reactive protein (CRP), erythrocyte sedimentation rate (ESR), creatine kinase (CK), triglycerides, and blood type, were collected. Laboratory data were obtained from the Clinical Data Warehouse at CUIMC after approval from the Tripartite Request Assessment Committee. Samples were obtained in the Emergency Department, at admission, and throughout the hospital stay, and were analyzed by the CUIMC Clinical Laboratories; residual samples that were no longer required for clinical purposes, were retrieved from the CUIMC Clinical Laboratories and banked for research studies. Clinical and demographic data, including name, medical record number (MRN), sex, date of birth, age, race, ethnicity, weight, body mass index, comorbidities (hypertension, diabetes mellitus, coronary artery disease, renal disease, hyperlipidemia, liver disease, lung disease), intubation/ventilator requirement, continuous veno-venous hemofiltration (CVVH) requirement, radiographically-confirmed thrombotic complications (deep vein thrombosis, pulmonary embolism, stroke), clotting of CVVH, hospitalization course (admission date, date of Emergency Department presentation, discharge date), mortality, and date of death were collected manually by reviewing the electronic medical record.

## Results

### General and Clinical Characteristics of Study Subjects

Patient demographic data are shown in Table 1. Briefly, COVID-19 (−) and COVID-19 (+) groups were similarly split across age, sex and racial/ethnic background. COVID-19 (−) and COVID-19 (+) patients presented with a similar prevalence of chronic conditions (hypertension, diabetes mellitus, chronic kidney disease) and both COVID-19 (−) and COVID-19 (+) patients demonstrated high median BMIs. The COVID-19 (+) patient median values for pro-inflammatory markers (C-reactive protein, ferritin, fibrinogen, and IL-6) were all increased by 1.5–2.0-fold greater than that observed in COVID-19 (−) patients. Inflammatory markers tracked with increased D-dimer levels. All clinical laboratory data that were obtained by request from patient charts are shown in Supplementary Table 1. An illness severity scoring system was not applied to patients included in this study. Nonetheless, comparisons between COVID-19 (−) and COVID (+) patients suggest a greater state of inflammation in COVID-19 (+) patients based on increased CRP (570% increase, *p* < 0.00010), IL-6 (179% increase, *p* < 0.014), ferritin (287% increase, *p* < 0.00010), fibrinogen (130%, *p* < 0.00010), and erythrocyte sedimentation rate (157%, *p* < 0.00010).

**Table 1:** Patient demographics and clinical characteristics.

**Patient characteristics**	**COVID-19 (−) (*n =* 288)**	**COVID-19 (+) (*n =* 543)**
**Age, median (range)**	62 (1.0–101)	63 (3.0–99)
**Sex**		
Female Male	*n =* 119 (43 %) *n =* 159 (57 %)	*n =* 203 (44 %) *n =* 264 (56 %)
**Race/Ethnicity**		
Asian African American/Black Caucasian/White Other Multi–racial Hispanic/Latino	*n =* 4 *n =* 56 *n =* 56 *n =* 13 *n =* 3 *n =* 104 (Black:7, White:21, other:18,	*n =* 3 *n =* 83 *n =* 49 *n =* 30 *n =* 1 *n =* 211 (Black:10, White: 35, other:32, Asian: 1, American Ind/Alaskan:1, Multiracial:1)
Declined	*n =* 52	*n =* 166
**Body mass index (kg/m** ^**2**^ **), median (range)**	25.5 (13.7–53.2)	28.0 (14.1–63)
**Ventilator**	*n =* 31 (11 %)	*n =* 67 (12 %)
**New thrombosis**		
New–DVT/PE New– Stroke	*n =* 22 (8.0 %) *n =* 7 (2.4 %)	*n =* 28 (5.0 %) *n =* 21 (4.0 %)
Chronic conditions^*^		
HTN DM CAD ESRD/CKD Cancer Stroke Hyperlipidemia Heart Failure Liver Disease Lung Disease	*n =* 134 (47 %) *n =* 81 (28 %) *n =* 48 (17 %) *n =* 35 (13 %) *n =* 22 (8.0 %) *n =* 21 (7.0 %) *n =* 34 (12 %) *n =* 39 (14 %) *n =* 13 (5.0 %) *n =* 50 (17 %)	*n =* 262 (48%) *n =* 184 (34 %) *n =* 54 (10 %) *n =* 69 (13 %) *n =* 38 (7.0%) *n =* 32 (6.0 %) *n =* 94 (17 %) *n =* 23 (4.0 %) *n =* 11 (2.0 %) *n =* 41 (8.0 %)
**Survivors** **Non–survivors**	*n =* 255 (88.5 %) *n =* 33 (11.5 %)	*n =* 433 (80 %) *n =* 110 (20 %)

* *History of chronic conditions: HTN, Hypertension, DM, Diabetes Mellitus; CAD, Coronary heart disease; ESRD/CKD, End-stage renal failure/chronic kidney disease. The percentage of patients per group for binary variables are indicated*.

### VWF/ADAMTS13 Axis Changes in Acutely Ill COVID-19 (−) and COVID-19 (+) Patients

**Figure 1:**
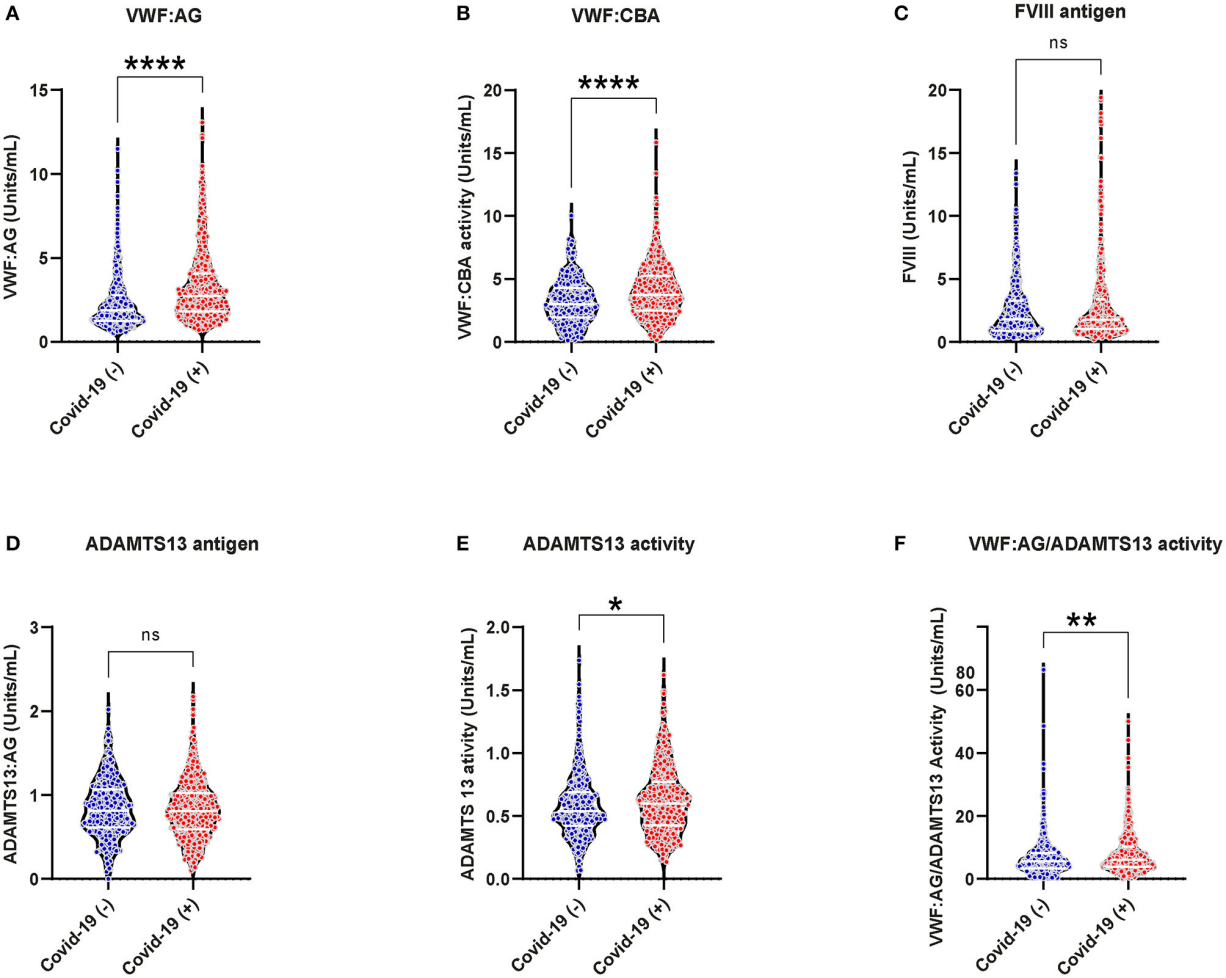
VWF/ADAMTS13 axis changes and coagulation in acutely ill COVID-19 (−) and (+) patients: **(A)** VWF antigen, 1.868 (IQR, 1.257–2.770) (−), 2.736 (IQR, 1.822–4.060) (+), *p* < 0.0001; **(B)** VWF collagen binding activity (9) 2.989 (IQR, 1.958–4.252) (−), 3.745 (IQR, 2.506–5.262) (+), *p* < 0.0001; **(C)** FVIII antigen 1.79 (IQR, 0.898–3.283) (−), 1.769 (IQR, 1.031–3.366) (+), *p* = 0.4154; **(D)** ADAMTS13 antigen 0.806 (IQR, 0.592–1.023) (−), 0.813 (IQR, 0.614–1.069) (+), *p* = 0.539; **(E)** ADAMTS13 activity 0.540 (IQR, 0.420–0.689) (−), 0.597 (IQR, 0.427–0.767) (+), *p* = 0.027; **(F)** VWF:AG/ADAMTS13 activity 5.567 (IQR, 3.352–8.245) (−), 6.051 (IQR, 3.824–10.17) (+), *p* < 0.0044. Datapoints indicate individual measurements, and *p*-values are from the Mann-Whitney analysis for comparison within groups. Values are presented as median and interquartile range (IQR, 25th−75th percentile) for continuous variables. ns, *P* > 0.05; *P ≤ 0.05; **P ≤ 0.01; ***P ≤ 0.001; ****P ≤ 0.0001.

Increased VWF:AG and activity were observed in both COVID-19 (+) and COVID-19 (−) patients (Figures 1A,B, VWF:AG reference range: ~ 0.5–2.0 U/mL). However, COVID-19 (+) patients demonstrated significantly higher VWF:AG and CBA levels compared to COVID-19 (−) patients (*p* < 0.0001). Respective median antigen and activity levels of VWF in the COVID-19 (+) group were 2.736 (IQR:1.822–4.060) and 3.745 (IQR:2.506–5.262) U/mL compared to 1.868 (IQR:1.257–2.770) and 2.989 (IQR:1.958–4.252) U/mL in the COVID-19 (−) group. A similar elevation of FVIII was observed in both COVID-19 (+) (Median:1.769 and IQR:1.031–3.366 U/mL) and COVID-19 (−) (Median:1.79 and IQR:0.898–3.283 U/mL) patients (Figure 1C, FVIII reference range: ~0.5–1.5 U/mL) with no significant differences between the groups. ADAMTS13 activity levels on the other hand were found to be lower in both COVID-19 (+) and COVID (−) patient groups when compared to the normal reference range (Figures 1D,E, normal ADAMTS13 activity levels: ≥ 0.5 U/mL). Specifically, ADAMTS13 activities in both groups were minimally decreased, but not lower than normal reference activity (50–160%). Respective median ADAMTS13 antigen and activity levels were 0.806 (IQR:0.592–1.023) and 0.597 (IQR:0.427–0.767) U/mL in COVID-19 (+) and 0.813 (IQR:0.614–1.069) and 0.54 (IQR:0.420–0.689) U/mL in COVID-19 (−) patients. The difference in ADAMTS13 activity levels between COVID-19 (+) and COVID-19 (−) patients was minimal, but statistically significant (*p* = 0.027). Subsequently, the VWF:AG/ADAMTS13 activity ratios in COVID-19 (+) patients (Median:6.051 and IQR:3.824–10.17) were significantly higher (*p* < 0.0001) than COVID-19 (−) patients (Median:5.567 and IQR:3.352–8.245) (Figure 1F). The data suggests that increased VWF:AG levels and VWF:CBA in plasmas of COVID-19 (+) patients occurred despite normal ADAMTS13 function. However, unlike in TTP the present data did not reveal thrombocytopenia in conjunction with increased VWF:AG levels and CBA in COVID-19 (+) patients (Supplementary Figure 1).

### Plasma Coagulation in Acutely Ill COVID-19 (−) and COVID-19 (+) Patients

**Figure 2:**
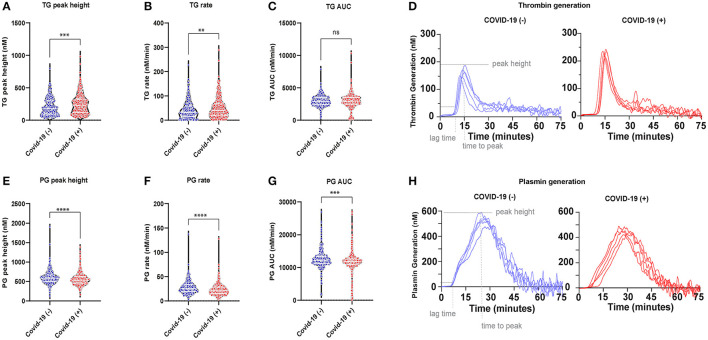
Plasma coagulation in acutely ill COVID-19 (−) and (+) patients: **(A)** Thrombin generation (TG) peak height 177.5 (IQR, 94.24–297.6) (−), 230.0 (IQR, 23.0–326.2) (+), *p* < 0.0005; **(B)** TG rate 32.66 (IQR, 15.78–57.69) (−), 40.38 (IQR, 20.39–67.33) (+), *p* = 0.0063; **(C)** TG AUC 2902 (IQR, 2442–3503) (−), 2,957 (IQR, 2,486–3,502) (+), *p* = 0.9187; **(D)** Representative TG curves from patient plasma; **(E)** Plasmin generation (PG) peak height 585.5 (IQR, 497.5–665.5) (−), 535.2 (IQR, 458.5–624.3) (+), *p* < 0.0001; **(F)** PG rate 25.20 (IQR, 19.26–33.69) (−), 20.97 (IQR, 15.31–28.57) (+), *p* < 0.0001; **(G)** PG AUC 12,239 (IQR, 11,277–13,369), 11,783 (IQR, 10,884–26,994), *p* = 0.0002; **(H)** Representative PG curves from patient plasma. Data points indicate individual measurements, and *p*-value comes from the Mann-Whitney analysis for comparison within groups. Values are presented as median and interquartile range (IQR, 25th–75th percentile) for continuous variables. TG, thrombin generation; PG, Plasmin generation. ns, *P* > 0.05; *P ≤ 0.05; **P ≤ 0.01; ***P ≤ 0.001; ****P ≤ 0.0001.

Thrombin generation increased, while plasmin generation decreased in the plasmas of COVID-19 (+) compared to COVID-19 (−) patients. An increased thrombin peak height and generation rate was observed with a simultaneously decreased plasmin peak height and generation rate in COVID-19 (+) patients (Figure 2). The median peak heights and thrombin generation rates in COVID-19 (+) patients were significantly increased by 25% [230.0 (IQR:123.0–326.2) nM] and 21% [40.38 (IQR:20.39–67.33) nM/min], respectively, compared to COVID-19 (−) patients (*p* < 0.01), (Figures 2A,B). The area under curve (AUC) values, however, were similar between COVID-19 (+) (2,957 nM/min) and COVID-19 (−) (2,902 nM/min) patients (Figure 2C). Representative thrombin generation curves from COVID-19 (+) and COVID-19 (−) patient plasmas are shown in Figure 2D. Plasmin peak height and generation rate were decreased by 9 and 18%, respectively, in COVID-19 (+) compared to COVID-19 (−) patients (*p* < 0.0001, Figures 2E,F). The median peak height and plasmin generation rate in COVID-19 (+) patients were 535.2 (IQR: 458.5–624.3) nM and 20.97 (IQR: 15.31–28.57) nM/min compared to 585.5 (IQR: 497.5–665.5) nM and 25.2 (IQR: 19.26–33.69) nM/min in COVID-19 (−) patients. Relative to healthy donor PPP, run under the same conditions (25), the median plasmin generation rates in COVID-19 (+) patients were ~40% lower. The AUC values were also significantly lower (*p* = 0.0002) in COVID-19 (+) patients (11,783 nM/min) compared to COVID-19 (−) patients (12,239 nM/min) (Figure 2G). Representative plasmin generation curves from COVID-19 (+) and COVID-19 (−) patient plasmas are shown in Figure 2H. These data demonstrate an increase in thrombin generation, suggesting a higher risk for thrombosis in COVID-19 (+) patients. Further, the observation of lower plasmin generation rates suggests an impaired fibrinolytic system in COVID-19 (+) patients. A similar distribution of platelet counts was observed in both COVID-19 (+) and COVID-19 (−) patients (Supplemental Figure 1).

### Age Dependent Differences in VWF/ADAMTS13 Axis and Plasma Coagulation Parameters

Increasing age is a contributing factor to illness severity and death from COVID-19 infection. The differences in VWF, ADAMTS13, thrombin generation, and plasmin generation parameters were evaluated in plasmas from COVID-19 (+) and COVID-19 (−) patients that were <65 and ≥65 years of age (Table 2).

**Table 2:** VWF, ADAMTS13, thrombin generation, and plasmin generation characteristics by age grouping.

**Parameters**	**< 65 years**			**≥ 65 years**			**<65 vs. ≥ 65 years**	**<65 vs. ≥ 65** **years**
	**COVID-19 (−)**	**COVID-19 (+)**	***P*-value**	**COVID-19 (−)**	**COVID-19 (+)**	***P*-value**	**COVID-19 (−)**	**COVID-19 (+)**
							***P*-value**	***P*-value**
ADAMTS13 Antigen (U/mL)	0.8805 (IQR, 0.6203–1.121)	0.8340 (IQR, 0.5950–1.084)	0.44	0.78 (IQR, 0.60–1.01)	0.7760 (IQR, 0.6033–1.013)	0.87	0.064	0.21
ADAMTS13 Activity (U/mL)	0.56 (IQR, 0.4483–0.7310)	0.6405 (IQR, 0.4743–0.8293)	0.0080	0.5085 (IQR, 0.3835–0.6673)	0.5460 (IQR, 0.4008–0.7203)	0.31	0.048	<0.00010
VWF: AG (U/mL)	1.690 (IQR, 1.236–2.395)	2.259 (IQR, 1.621–3.296)	<0.00010	2.019 (IQR, 1.303–3.119)	3.128 (IQR, 2.118–4.630)	<0.00010	0.050	<0.00010
VWF: CBA (U/mL)	2.875 (IQR, 1.591–4.115)	3.433 (IQR, 2.235–4.576)	0.0020	3.281 (IQR, 2.028–4.631)	4.147 (IQR, 2.742–5.597)	0.00010	0.021	<0.00010
VWF:AG/ADAMTS13 activity	4.827 (IQR, 2.857–7.709)	4.984 (IQR, 3.316–7.988)	0.27	5.923 (IQR, 4.108–8.843)	7.349 (IQR, 4.509–12.12)	0.0075	0.0065	<0.00010
FVIII (U/mL)	1.48 (0.77–3.32)	1.61 (0.84–2.88)	0.716	2.34 (1.30–3.87)	1.87 (1.17–3.61)	0.275	0.0021	0.0016
TG Peak Height (nM)	167.1 (IQR, 87.56–292.0)	211.4 (IQR, 101.1–306.0)	0.055	178.0 (IQR, 103.1–292.0)	247.4 IQR, (144.7–340.1)	0.00070	0.34	0.016
TG Rate (nM/min)	29.06 (IQR, 14.37–55.25)	35.63 (IQR, 17.15–63.08)	0.070	36.22 (IQR, 16.60–56.54)	44.76 (IQR, 21.42–69.19)	0.020	0.24	0.087
PG Peak Height (nM)	570.8 (IQR, 492.6–679.2)	562.6 (IQR, 464.8–643.6)	0.18	581.0 (IQR, 487.9–638.5)	519.8 (IQR, 446.6–602.2)	0.00060	0.75	0.0067
PG Rate (nM/min)	24.59 (IQR, 18.84–33.37)	22.29 (IQR, 16.18–29.65)	0.041	25.11 (IQR, 18.67– 32.48)	19.25 (IQR, 14.78– 25.99)	<0.00010	0.81	0.015

*AG, antigen; CBA, Collagen binding activity*.

In patients <65 years of age, significant increases in median VWF:AG and VWF:CBA levels in COVID-19 (+) patients were observed. Specifically, the median VWF:AG, and VWF:CBA levels in the COVID-19 (+) group were increased by 28.8% and 17%, respectively, compared to the COVID-19 (−) group (*p* < 0.0001; *p* = 0.002) (Table 2). The increase in VWF levels and binding activity are consistent with endothelial dysfunction in patients <65 years of age. Despite the changes in VWF, no changes in VWF/ADAMTS13 activity were observed. Among patients <65 years of age, median plasmin generation rates reached statistical significance (*p* < 0.05). The median plasmin generation rates in COVID-19 (+) patients decreased by 9% compared to COVID-19 (−) patients (*p* = 0.041). Despite the changes in plasmin generation, no differences in thrombin generation were observed.

Among patients ≥65 years of age, no significant differences in ADAMTS13, ADAMTS13 activity or FVIII levels were observed between COVID-19 (+) or COVID-19 (−) groupings. On the other hand, significantly elevated VWF:AG, VWF:CBA and VWF:AG/ADAMTS13 activity ratios were observed in COVID-19 (+) patients (Table 2). Specifically, in the COVID-19 (+) group, median VWF:AG, VWF: CBA and VWF:AG/ADAMTS13 activity ratios increased (by 43, 23, and 21.5%, respectively) compared to the COVID-19 (−) group (Table 2). Among thrombin and plasmin parameters, elevated coagulation and decreased fibrinolysis was observed in COVID-19 (+) patients. Within this group, median thrombin peak heights and generation rates increased by 32.6% (*p* = 0.0007) and 21% (*p* = 0.02), respectively. Conversely, median plasmin peak heights and generation rates decreased by 11% (*p* = 0.0006) and 26% (*p* < 0.0001), respectively, compared to COVID-19 (−) patients (Table 2). Comparisons between the two age groups within the COVID-19 (+) patients (Table 2) indicates that patients ≥65 years of age have a reduced plasma ADAMTS13 activity (remaining in the reference range), as well as increased VWF:AG, VWF:CBA, and VWF/ADAMTS13 activity ratio. Further, thrombin and plasmin generation parameters were increased and decreased, respectively. Comparisons between <65 and ≥ 65-year-old individuals are also provided for the COVID-19 (−) patient group (Table 2). A similar distribution of platelet count was observed in both COVID-19 (+) and COVID-19 (−) across patients grouped as <65 and ≥65 years of age (Supplementary Figure 1).

This data indicates that a main difference between younger and older COVID-19 (+) patients evaluated in the present study was increased VWF:AG levels and activities in older patients. More importantly the age of COVID-19 (+) patients defined a risk factor for promoting hemostasis and impairing fibrinolysis based on enhanced thrombin generation and impaired plasmin generation, respectively.

### BMI Dependent Differences in VWF/ADAMTS13 Axis and Plasma Coagulation Parameters

A BMI greater than normal (> 25 kg/m^2^) represents an important risk for COVID-19 illness severity. To assess the effect of BMI on VWF/ADAMTS13 axis changes, we grouped patients based on CDC guidelines into four BMI categories: <18.5, 18.5–24.9, 25–29.9, >30 (kg/m^2^). Within underweight and normal healthy BMI grouping, VWF:AG, VWF: CBA, ADAMTS13 antigen, and ADAMTS13 activity levels did not differ based on COVID-19 (−) or COVID-19 (+) status. VWF:AG, VWF:CBA and VWF:AG/ADAMTS13 activity were significantly increased in COVID-19 (+) patients within the overweight (25–29.9 kg/m^2^) and obese (>30 kg/m^2^) BMI groupings (Table 3). However, ADAMTS13 levels and activities were unchanged within the overweight (25–29.9 kg/m^2^) and obese (>30 kg/m^2^) BMI groupings regardless of COVID-19 status (Table 3). Plasma coagulation and fibrinolysis parameters measured by simultaneous thrombin and plasmin generation showed a significant inhibition of fibrinolysis in the plasmas of obese (>30 kg/m^2^, BMI) COVID-19 (+) patients. Median plasmin generation rates decreased by ~25% in the plasma of COVID-19 (+) obese patients. Comparisons between BMI categorization in the COVID-19 (+) group demonstrated no significant differences in assayed parameters from plasmas collected at hospital presentation or admission. A similar distribution of platelet counts was observed in both COVID-19 (+) and COVID-19 (−) across patient BMI groupings (Supplementary Figure 1).

**Table 3:** VWF, ADAMTS13, thrombin generation, and plasmin generation characteristics by BMI groupings.

**Parameters**	**BMI <18.5**			**BMI 18.5–24.9**			**BMI 25–29.9**			**BMI >30**		
	**COVID-19 (−)**	**COVID-19 (+)**	***P*-value**	**COVID-19 (−)**	**COVID-19 (+)**	***P*-value**	**COVID-19 (−)**	**COVID-19 (+)**	***P*-value**	**COVID-19 (−)**	**COVID-19 (+)**	***P*-value**
ADAMTS13 Antigen (U/mL)	0.6540 (IQR, 0.5328–0.9453)	0.8530 (IQR, 0.4920–1.479)	0.43	0.7660 (IQR, 0.5750–0.9800)	0.7350 (IQR, 0.5030–0.9335)	0.30	0.9330 (IQR, 0.6360–1.148)	0.7950 (IQR,0.5883–1.002)	0.11	0.8510 (IQR, 0.6425–1.125)	0.8210 (IQR, 0.5660–1.027)	0.16
ADAMTS13 Activity (U/mL)	0.4785 (IQR, 0.4195–0.5963)	0.7580 (IQR, 0.5818–1.006)	0.054	0.5030 (IQR, 0.3950–0.6000)	0.6000 (IQR, 0.4298–0.7278)	0.032	0.6100 (IQR, 0.5090–0.7940)	0.6070 (IQR, 0.4190–0.7860)	0.27	0.5600 (IQR, 0.4250–0.7955)	0.5645 (IQR, 0.4030–0.7683)	0.40
VWF: AG (U/mL)	2.073 (IQR, 2.587–1.319)	3.443 (IQR, 4.877–1.819)	0.60	2.069 (IQR, 3.066–1.389)	2.725 (IQR, 4.414–1.843)	0.0029	1.661 (IQR, 2.944–1.252)	3.012 (IQR, 3.888–2.157)	<0.00010	1.855 (IQR, 2.943–1.268)	3.023 (IQR, 4.337–2.186)	<0.00010
VWF: CBA (U/mL)	2.821 (IQR, 2.501–4.313)	3.445 (IQR, 1.541–5.838)	0.60	3.355 (IQR, 4.378–2.092)	3.519 (IQR, 5.074–2.423)	0.17	2.945 (IQR, 4.161–2.097)	4.182 (IQR, 5.151–3.277)	0.00020	3.044 (IQR, 4.228–2.137)	4.292 (IQR, 5.688–2.884)	<0.00010
VWF:AG/ADAMTS13 activity	6.120 (IQR, 7.799–4.586)	3.315 (IQR−8.694–2.592)	0.18	6.138 (IQR, 9.228–3.680)	5.815 (IQR, 9.805–4.258)	0.79	4.752 (IQR, 6.759–3.386)	7.328 (IQR, 10.06–4.954)	<0.00010	5.131 (IQR, 7.025–3.172)	7.157 (IQR, 12.83–4.223)	0.0015
FVIII (U/mL)	3.444 (IQR, 2.025–5.537)	1.192 (IQR, 0.7350–4.455)	0.0674	2.172 (IQR, 1.262–4.063)	1.862 (IQR, 1.192–3.599	0.7619	1.927 (IQR, 0.7975–3.333	1.754 (IQR, 1.087–3.203	0.9008	1.829 (IQR, 0.7015–3.739	1.717 (IQR, 0.9930–3.231	0.7590
TG Peak Height (nM)	232.2 (IQR, 68.22–339.2	213.7 (IQR, 63.73–470.4	0.76	193.4 (IQR, 88.40–287.9)	226.9 (IQR, 127.3) 297.8	0.14	177.9 (IQR, 122.0–339.6	246.9 (IQR, 101.1–337.4	0.52	202.6 (IQR, 111.0–323.5	255.4 (IQR, 153.4–354.8	0.071
TG Rate (nM/min)	36.73 (IQR, 14.66–66.79)	46.58 (IQR, 12.75–107.5)	0.56	33.15 (IQR, 14.11–54.96)	37.48 (IQR, 18.50–59.06)	0.36	35.22 (IQR, 18.19–66.09)	46.16 (IQR, 17.30–71.33)	0.61	38.11 (IQR, 18.23–66.03	45.99 (IQR, 25.04–78.96	0.097
PG Peak Height (nM)	612.2 (IQR, 479.1–877.0)	505.0 (IQR, 459.8–624.6)	0.21	578.7(IQR, 498.6–636.6)	520.8 (IQR, 426.2–604.6)	0.013	593.2 (IQR, 504.8–641.9)	545.0 (IQR, 487.7–647.7)	0.40	585.0 (IQR, 527.7–682.0)	537.0 (IQR, 458.7–638.1)	0.026
PG Rate (nM/min)	32.48 (IQR, 19.32–42.19)	24.24 (IQR, 15.06–30.71)	0.12	22.75 (IQR, 16.68–28.41)	19.68 (IQR, 14.27–27.63)	0.11	23.87 (IQR, 18.15–30.68)	22.65 (IQR, 17.64–28.63)	0.31	26.36 (IQR, 18.49–32.43)	20.77 (IQR, 14.88–26.62)	0.0058

*AG, antigen; CBA, Collagen binding activity*.

### VWF/ADAMTS13 Axis Changes and Plasma Coagulation Parameters in Survivors and Non-survivors

The VWF/ADAMTS13 axis as well as plasma hemostasis and fibrinolysis were compared within the COVID-19 (−) and COVID-19 (+) groups to understand the differences in VWF/ADAMTS13 axis and plasma coagulopathy between surviving and non-surviving patients (Table 4). At the time of the initial blood draw, hospitalized COVID-19 (−) patients who ultimately did not survive their illness demonstrated significantly (*p* < 0.05) higher VWF levels and collagen binding activity as well as higher FVIII levels compared to COVID-19 (−) patients who survived their illness. The same parameters were also significantly (*p* < 0.05) increased in non-surviving COVID-19 (+) patients; however, survival was increased by 3.5-fold (*p* < 0.0001) in the COVID-19 (+) group compared to the COVID-19 (−) group.

**Table 4:** VWF, ADAMTS13, thrombin generation, and plasmin generation characteristics by survivors and non–survivors in COVID-19 positive and negative groups.

**Parameters**	**COVID-19 (−)**			**COVID-19 (+)**		
	**Survivors**	**Non–survivors**	***P*-value**	**Survivors**	**Non–survivors**	***P*-value**
ADAMTS13 Antigen (U/mL)	0.8320 (IQR, 0.6285–1.081)	0.6670 (IQR, 0.4230–1.019)	0.067	0.8140 (IQR, 0.5688–1.014)	0.6990 (IQR, 0.5310–0.8870)	0.013
ADAMTS13 Activity (U/mL)	0.5560 (IQR, 0.4445–0.7070)	0.4000 (IQR, 0.3010–0.5720)	0.00020	0.5930 (IQR, 0.4150–0.7620)	0.4870 (IQR, 0.3563–0.6778)	0.0095
VWF: AG (U/mL)	2.937 (IQR, 1.920–4.191)	3.972 (IQR, 2.409–5.012)	0.0010	2.561 (IQR, 1.782–3.877)	3.327 (IQR, 2.366–5.838)	<0.00010
VWF: CBA (U/mL)	1.681 (IQR, 1.241–2.554)	2.268 (IQR, 1.799–4.649)	0.026	3.640 (IQR, 2.523–5.108)	4.506 (IQR, 3.286–5.900)	0.00040
VWF:AG/ADAMTS13 activity	5.185 (IQR, 3.212–7.886)	7.558 (IQR, 5.027–13.73)	0.00050	6.275 (IQR, 3.817–10.08)	8.105 (IQR, 5.043–13.44)	0.00010
FVIII (U/mL)	1.711 (IQR, 0.8755–3.392	2.740 (IQR, 2.028–4.888)	0.0101	1.678 (IQR, 0.9620–2.949)	2.706 (IQR, 1.561–5.594)	<0.0001
TG Peak Height (nM)	174.8 (IQR, 96.79–295.3)	176.9 (IQR, 83.24–252.7)	0.52	227.2 (IQR, 118.5–315.2)	227.7 (IQR, 119.5–334.8)	0.81
TG Rate (nM/min)	31.14 (IQR, 16.01–57.06)	39.31 (IQR, 10.26–56.15)	0.97	40.26 (IQR, 20.24–69.04)	39.55 (IQR, 19.63–64.72)	0.72
PG Peak Height (nM)	575.7 (IQR, 495.2–656.1)	568.0 (IQR, 443.3–546.4)	0.26	537.9 (IQR, 458.1–624.3)	506.1 (IQR, 406.5–576.9)	0.0064
PG Rate (nM/min)	24.78 (IQR, 19.30–33.50)	23.92 (IQR, 13.07–31.78)	0.12	21.53 (IQR, 14.78–28.85)	17.99 (IQR, 13.34–24.15)	0.0040

*AG, antigen; CBA, Collagen binding activity*.

## Discussion

COVID-19 infected patients are at greater risk for venous and arterial thrombosis, particularly once the severity of disease requires intensive care (5, 28–30). Several studies identify important links between metabolic and protein changes that indicate up-regulated coagulation linked to inflammation and complement and offer unique insight into the relevant changes in COVID-19 coagulation omics (31–33). However, to our knowledge, no study has specifically focused on VWF/ADAMTS13 axis changes of coagulation combined with thrombin and plasmin generation in COVID-19 (−) or COVID-19 (+) patient cohorts at the time of hospital presentation and admission. Further, the present analysis focuses on plasma coagulation parameters in these two cohorts and then, more specifically, based on aging or BMI categorization and finally on changes in the VWF/ADAMTS13 axis and plasma coagulation in survival. Here we evaluate VWF/ADAMTS13 axis changes that suggest an early endothelial-based coagulopathy along with imbalanced plasma thrombin and plasmin generation.

Microvascular thrombosis caused by endothelial dysregulation is tied to immune activation and is an important pathophysiological response to COVID-19 infection (34). A review of autopsy findings identified that ~60% of deceased COVID-19 patients evaluated demonstrate microvascular thrombosis (35). Microthrombi are primarily observed in the lungs (~75% of cases), but also in the kidneys, liver, and heart (35). Within lung tissue, histopathology and immunohistochemistry analyses provide evidence of widespread primary pathology across alveolar sites and the peripheral lung vasculature, including pre- and post-capillary pulmonary vessels (34, 36). The microthrombi described in small pulmonary arteries and veins demonstrate immunoreactivity for platelets and megakaryocytes (i.e., CD61), fibrin, VWF, and lymphocytes (i.e., CD4, CD8) (36). Interestingly, the localized pulmonary coagulopathy in COVID-19 pneumonia is more pronounced than that in influenza or bacterial pneumonia, and demonstrates an upregulated gene signature consistent with hypoxia-induced intussusceptive “splitting” angiogenesis (34). Platelet- and VWF-rich thrombi demonstrate greater resistance to thrombolytic therapies (37, 38) suggesting that treatment options are limited after established microvascular thrombosis in severe COVID-19 infection.

These observations of increased microvascular thrombosis caused by endothelial dysregulation influenced studies on the contributions of VWF and ADAMTS13 across a range of pro-thrombotic processes and COVID-19 disease severities (9, 39, 40). VWF is an acute-phase reactant and its secretion from endothelial cells increases in response to various stimuli, including shear stress and inflammation (41). During the inflammatory activation associated with COVID-19, the vascular imbalance of VWF and ADAMTS13 favors an elevated VWF:AG/ADAMTS13 activity ratio; this shift is implicated in localized endothelial dysfunction of COVID-19 infection (10–13). A close relationship with the VWF/ADAMTS13 axis and hospitalized COVID-19 (+) patients disease severity (low, intermediate, and high) is identified (11). This study also reports on VWF multimer accumulation in the plasmas of COVID-19 (+) patients suggesting a relationship between endothelial coagulation and COVID-19 disease severity. Two additional studies specifically identify the upper limits of VWF:AG levels (4.23-fold greater than normal) (12) and collagen binding activity (4.46-fold greater than normal) as predictors of mortality (13).

Our observations suggest that VWF:AG is increased in the plasma of both COVID-19 (−) and COVID-19 (+) patients at hospital presentation and admission. However, VWF:AG levels exceed the reference range (0.5–2 U/mL) and VWF collagen binding activity is significantly increased in COVID-19 (+) patients. Factor VIII levels were not found to be changed at the time of hospital presentation in the COVID-19 (+) patient plasmas analyzed in this study. Despite elevated VWF function in COVID-19 (+) patients' plasma, only mild changes in ADAMTS13 levels or activity are observed. Our rationale to measure ADAMTS13 levels was based on reports of ADAMTS13 antigen and activity decreases in other infections, including bacterial sepsis (42), and in viral infection-induced secondary TTP due to ADAMTS13 specific IgG inhibitor production (43). Nonetheless, the ratio of VWF:AG to ADAMTS13 activity does increase because of the higher VWF:AG levels. These observations differ from those observed in diseases of endothelial micro-thrombotic origin. For example, TTP is characterized by loss of ADAMTS13 function, thrombocytopenia, and schistocytosis (44). In the present study, COVID-19 (+) patient plasma showed normal ADAMTS13 functional activity (≥50%) and normal platelet levels (~250 × 10^9^/L), consistent with prior studies of COVID-19 disease progression and severity (10, 13). Although not widespread across the spectrum of COVID-19-induced coagulopathy, some reports include case descriptions of TTP during ongoing infection; that is, microangiopathic hemolytic anemia with schistocytes and thrombocytopenia (19, 45).

COVID-19 disease outcome is widely reported to be affected by age and underlying comorbidities. For example, patients of increasing age and, independently, of increasing BMI are reported to be at greater risk for thrombosis based on underlying systemic organ functional decline and the likelihood of comorbidities (46, 47). In addition, comorbid states consistent with increasing age and increased BMI track with COVID-19 disease progression (48–50). Specifically, the median age in the present study was 62 and 63 years of age in the COVID-19 (−) and COVID-19 (+) cohorts, respectively, and, within the two groups, the patients were almost equally split between individuals younger and older than 65. Based on our current data with COVID-19 (−) and COVID-19 (+) patients, there was a clear age-dependent effect (i.e., ≥65) on VFW:AG, VWF collagen binding activity, and the VWF:AG/ADAMTS13 activity ratio, suggesting an enhanced potential for endothelial coagulopathy. A shift toward increased thrombin generation and decreased plasmin generation was observed in COVID-19 (+) patients >65 years of age in the present study, suggesting an increased risk for hemostasis and impaired fibrinolysis.

Assessment of coagulation in COVID-19 using viscoelastic coagulation tests (e.g., TEG and ROTEM) offers an important insight into the potential for hemostasis and the likelihood for effective fibrin clot lysis in whole blood and platelet rich plasma (51). These assays can be performed at bedside, and are potentially useful in the diagnosis and treatment of COVID-19-induced coagulopathy (27, 52). Several studies that utilize viscoelastic coagulation tests demonstrate elevated clot strength in COVID-19 infection (34, 53, 54). However, viscoelastic tests do not specifically determine the amount of thrombin or plasmin produced in the patient's sample, and the sensitivity of viscoelastic tests to detect fibrinolysis remains controversial (55). For example, in several cases of COVID-19 coagulopathy, analysis by ROTEM suggested that fibrinolysis is completely inhibited (56). However, we do not observe complete inhibition of plasmin generation in the plasma samples evaluated in the study described here. Our study employed a research-based simultaneous thrombin and plasmin generation enzymatic assay to assess the potential for hemostasis and fibrinolysis in PPP (26, 57–59). An important feature of this approach allows for an improved understanding of the rate of thrombin generation, but also an accurate assessment of plasmin generation rates and functional fibrinolysis within a sample. Analysis of 288 COVID-19 (−) and 543 COVID-19 (+) plasma samples obtained at the time of hospital presentation and admission suggested increased thrombogenic potential/ dysregulated hemostasis based on significantly greater thrombin peak heights and generation rates in COVID-19 (+) patients. In addition, impaired fibrinolysis was suggested by identifying significantly lower plasmin peak heights and generation rates in COVID-19 (+) patient samples (60). Interestingly, patients ≥65 years of age, which comprised ~50% of the patient population studied, accounted for the highest thrombin generation rates and the lowest plasmin generation rates. Unexpectedly, neither overweight nor obese patients demonstrated increased thrombin generation, and only obese patients (i.e., ≥30 kg/m^2^) demonstrated significantly lower plasmin generation. Collectively, this may indicate that age is one of the most important additive risk factors for dysregulated hemostasis in COVID-19 infection. This is not to say that all patients of increasing age develop thrombosis during COVID-19 infection, and these observations are likely due to existing comorbidities; for example, an aging endothelium and lower organ function naturally occurs over time. Finally, median D-dimer levels were increased in both COVID-19 (−) and (+) patients, but to a greater extent in the latter. However, active thrombosis was not ubiquitous in the patient cohorts described in our study, suggesting that ongoing fibrinolysis, unrelated to clot degradation, is relevant in COVID-19 (61).

The VWF/ADAMTS13 axis is significantly imbalanced in favor of higher VWF levels and activity and lower ADAMTS13 levels and activity in both acutely ill COVID-19 (−) and COVID-19 (+) non-survivors at the time of hospital admission. The VWF:AG/ADAMTS13 activity ratio was increased by 32 vs. 23% in COVID-19 (−) and COVID-19 (+) non-surviving patients, respectively. The samples in this study were analyzed in plasma from blood drawn at the time of hospital presentation or early after hospitalization and did not focus on temporal changes involved in disease progression. The most distinct difference between COVID-19 (+) and COVID-19 (−) non-survivors was a decrease in plasmin generation in COVID-19 (+) patients. This observation may suggest a COVID-19-induced impairment in fibrinolysis mediated by plasminogen activator inhibitor 1 (PAI-1) (62, 63), consistent with greater expression of the inhibitor in adipose tissue (64) and endothelium (65).

The present study defines VWF/ADAMTS13 axis parameters as markers of endothelial dysfunction, along with thrombin and plasmin generation as predictors of thrombosis and fibrinolysis, based on two important risk factors known to predict poor outcome in COVID-19 infection: increased age (66) and obesity (48). However, this study does have several acknowledged limitations. First, although most patients were admitted to inpatient care in both the COVID-19 (−) and COVID-19 (+) groups, some patients had blood draws in the Emergency Department and were discharged to home; therefore, only the sickest COVID-19 (−) patients are represented in this study. Second, hospitalized COVID-19 (−) and COVID-19 (+) patients demonstrate considerable differences in pathophysiology and not all co-morbidities could be captured based on the number of patients in need of care. Notably, COVID-19 (+) patients evaluated in this study demonstrated increased markers of inflammation as compared to COVID-19 (−) patients. Third, BMI values were not available for all patients. In the COVID-19 (−) group, 206 of 288 (72%) patient BMIs were available; in the COVID-19 (+) group 478 of 543 (88%) of patient BMIs were available. Fourth, the simultaneous measurement of thrombin and plasmin is a research-based methodological approach to assess thrombin and plasmin function and standardized reference values across laboratories are not available. Therefore, data can only be compared when evaluated across study groups. Nonetheless, this does not diminish the potential relevance of VWF/ADAMTS13 axis parameters, and of plasma thrombin and plasmin generation parameters, regarding the COVID-19 (+) patients evaluated in this study.

**Figure 3:**
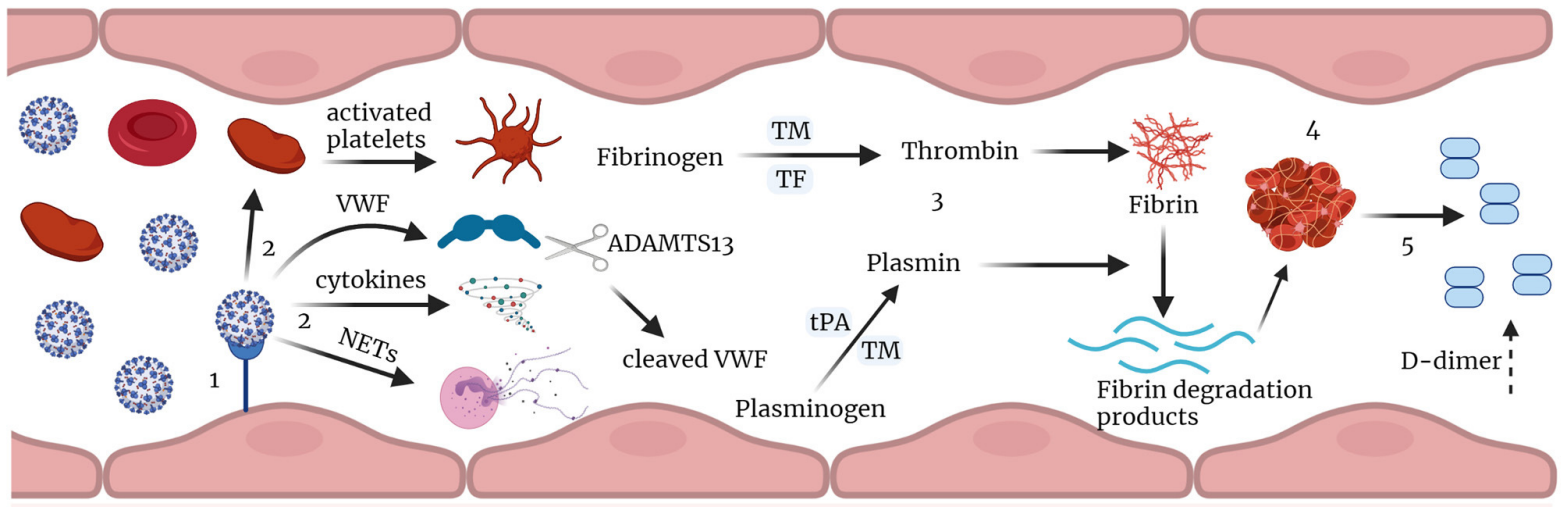
Immune-thrombosis in COVID-19: endothelial and plasma coagulation: (1) Viral infection leading to endothelial damage and inflammation. (2) this leads to increased expression of cytokines, and procoagulant factors like VWF, activation of platelets and neutrophil extracellular traps (NETs) release; VWF is cleaved by ADAMTS13. (3) increased thrombin and plasmin generation potential in presence of thrombomodulin leading to increase in fibrin degradation products and D-dimer. (4) thrombus formation and (5) subsequent fibrin degradation that results in increased D-dimer in Covid-19. TM, thrombomodulin; tPA, tissue plasminogen activator; TF, tissue factor; VWF, von Willebrand factor; NETs, neutrophil extracellular traps (Figure created with Biorender.com).

In conclusion, these data are consistent with early signs of endothelial damage that may reflect the pulmonary immune-thrombosis seen with COVID-19 (Schematic Figure 3). The median VWF:AG level, VWF: CBA, and VWF:AG/ADAMTS13 activity ratio were all increased in COVID-19 (+) patients, as compared to the acutely ill COVID-19 (−) cohort. However, changes in median ADAMTS13 levels and activity were not observed. Similarly, median platelet levels were unchanged, and thrombocytopenia was not a consistently seen clinical finding, ruling out typical, and likely atypical, TMA. Furthermore, increased plasma coagulation, as determined by thrombin and plasmin generation, suggests the potential for dysregulated hemostasis in COVID-19 infection. This latter observation was almost exclusively weighted toward patients ≥65 years of age and surprisingly less relevant in overweight and obese COVID-19 (+) patients. Surprisingly, no differences in VWF/ADAMTS13 axis parameters were observed in critically ill COVID-19 (−) versus COVID-19 (+) non-survivors, while a significant imbalance, favoring endothelial coagulopathy was observed between surviving and non-surviving patients in each cohort. This retrospective analysis of acutely ill COVID-19 (−) and COVID-19 (+) patients suggests VWF/ADAMTS13 axis parameters, along with thrombin and plasmin generation, are relevant coagulation parameters to measure in early COVID-19 infection. The assessment of thrombin generation, but more specifically plasmin generation offers critical insight into impaired fibrinolysis not easily obtained by viscoelastic tests.

## Data Availability Statement

The original contributions presented in the study are included in the article/Supplementary Material, further inquiries can be directed to the corresponding authors.

## Ethics Statement

This study was approved by the Institutional Review Board of Columbia University Irving Medical Center (CUIMC) (Protocol Number AAAT0680). This study was conducted under a waiver of informed consent.

## Author Contributions

KTh, UK, IA, TT, SK, AW, JV, AD'A, SLS, ROF, and PB contributed to planning and design of the study and to the analysis and interpretation of data. KTh, UK, ROF, and PB drafted the manuscript. All authors critically revised the manuscript for content and approved the final version.

## Funding

This research was supported by funds from the RM1GM131968 (AD'A) from the National Institute of General and Medical Sciences, R01HL146442 (AD'A), R01HL149714 (AD'A), R01HL148151 (SLS, AD'A, JZ, ROF, and DR), R21HL150032 (AD'A), R01HL156526, R01HL159862 (PB and DR), and K23HL151901 from the National Heart, Lung and Blood Institute, W81XWH-20-PRMRP-IIRA-COV (DR) DoD.

## Conflict of Interest

IA is a consultant for Pharmacosmos Therapeutics. DR receives consulting fees from Portola Pharmaceuticals. AD'A and TN are founders of Omix Technologies Inc and Altis Biosciences LLC. AD'A and SLS are consultants for Hemanext Inc. SLS is also a consultant for Tioma, Inc. and TCIP, Inc., and the Executive Director of the Worldwide Initiative for Rh Disease Eradication WIRhE. AD'A is a consultant for FORMA LLC. The remaining authors declare that the research was conducted in the absence of any commercial or financial relationships that could be construed as a potential conflict of interest.

## Publisher's Note

All claims expressed in this article are solely those of the authors and do not necessarily represent those of their affiliated organizations, or those of the publisher, the editors and the reviewers. Any product that may be evaluated in this article, or claim that may be made by its manufacturer, is not guaranteed or endorsed by the publisher.

## Supplementary Material

Supplementary Figure 1 Platelet levels in COVID-19 (−) and COVID-19 (+) patients: **(A)** Total platelets 222(IQR, 168–293.8) (−), 220(IQR, 152–287) (+), *p* = 0.3977; **(B)** Platelets levels by age groups: <65: 233(IQR, 177–313) (−); 232(IQR, 173–299.5) (+), *p* = 0.6889; >65: 214(IQR, 162–279) (−), 227.5(IQR, 164.8–287) (+), *p* = 0.3809; Platelet levels by BMI **(C)** BMI <18.5: 164.5(IQR, 105.5–232) (−), 188(IQR, 124–325.5) (+), *p* = 0.5079; **(D)** BMI 18.5–24.9: 220(IQR, 156.8–297) (−), 211.5(IQR, 148.8–271.3) (+), *p* = 0.3904; **(E)** BMI 25–29.9: 216(IQR, 184.8–299.3) (−), 252(IQR, 177.5–303) (+), *p* = 0.6124; **(F)** BMI >30: 241.5(170–341.5) (−), 238(179.8–300) (+), *p* = 0.9451. Datapoints indicate individual measurements, and *p*-values were obtained from the Mann-Whitney analysis for comparison within groups. Values are presented as median and interquartile range (IQR, 25th–75th percentile) for continuous variables.
