# Supplementary figures and images for: The Impact of Age and BMI on the VWF/ADAMTS13 Axis and Simultaneous Thrombin and Plasmin Generation in Hospitalized COVID-19 Patients
Source: Front Med (Lausanne). 2022 Jan 10;8:817305. doi: 10.3389/fmed.2021.817305 (PMC8786628; doi:10.3389/fmed.2021.817305)

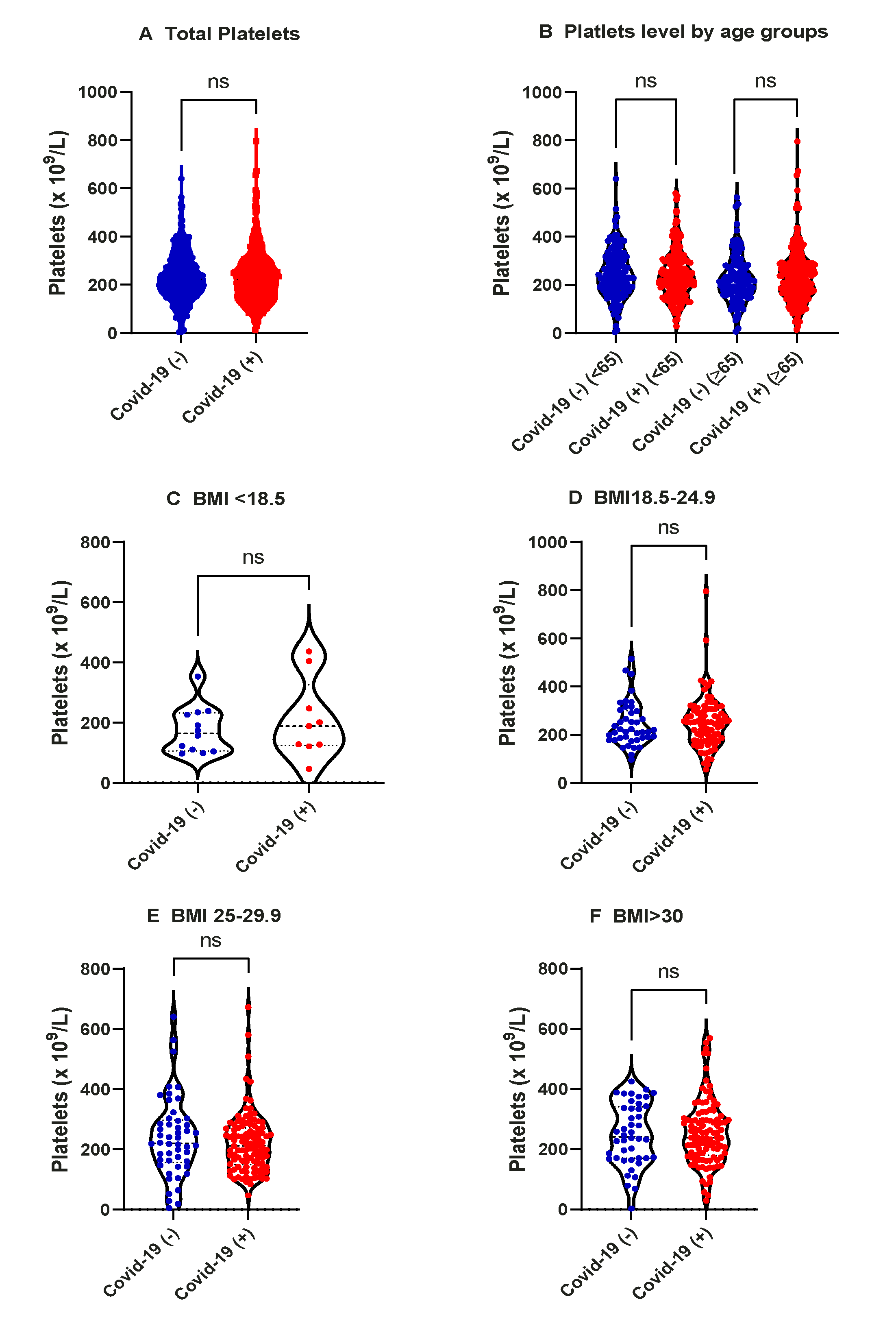

Supplement: Supplementary Figure 1 — Platelet levels in COVID-19 (−) and COVID-19 (+) patients: (A) Total platelets 222(IQR, 168–293.8) (−), 220(IQR, 152–287) (+), p = 0.3977; (B) Platelets levels by age groups: <65: 233(IQR, 177–313) (−); 232(IQR, 173–299.5) (+), p = 0.6889; >65: 214(IQR, 162–279) (−), 227.5(IQR, 164.8–287) (+), p = 0.3809; Platelet levels by BMI (C) BMI <18.5: 164.5(IQR, 105.5–232) (−), 188(IQR, 124–325.5) (+), p = 0.5079; (D) BMI 18.5–24.9: 220(IQR, 156.8–297) (−), 211.5(IQR, 148.8–271.3) (+), p = 0.3904; (E) BMI 25–29.9: 216(IQR, 184.8–299.3) (−), 252(IQR, 177.5–303) (+), p = 0.6124; (F) BMI >30: 241.5(170–341.5) (−), 238(179.8–300) (+), p = 0.9451. Datapoints indicate individual measurements, and p-values were obtained from the Mann-Whitney analysis for comparison within groups. Values are presented as median and interquartile range (IQR, 25th–75th percentile) for continuous variables. [file Image_1.tif]
