# Supplementary material for: The Impact of Age and BMI on the VWF/ADAMTS13 Axis and Simultaneous Thrombin and Plasmin Generation in Hospitalized COVID-19 Patients
Source: Front Med (Lausanne). 2022 Jan 10;8:817305. doi: 10.3389/fmed.2021.817305 (PMC8786628; doi:10.3389/fmed.2021.817305)
Supplement: Supplementary file 2 [file Data_Sheet_1.docx]

**Supplementary Table 1** Clinical laboratory parameters COVID-19 (-) and COVID-19 (+) patient groups

| **Patient characteristics** | **COVID-19 (-)** | | **COVID-19 (+)** | | **P Value** |
| --- | --- | --- | --- | --- | --- |
| **Complete blood count** | | | | | |
| **Red cell distribution width (%)** | 14.60 (IQR, 13.40-16.28) | n=272 | 14.40 (IQR, 13.20-16.50) | n=486 | 0.26 |
| **Erythrocyte sedimentation rate (mm/hr)** | 47.00 (IQR, 24.0-89.0) | n=103 | 74.0 (IQR, 48.75-96.0) | n=310 | <0.00010 |
| **Red blood cells (x10(6)/uL)** | 4.260 (IQR, 3.475-4.698) | n=272 | 4.120 (IQR, 3.438-4.740) | n=486 | 0.93 |
| **Reticulocytes (%)** | 1.720 (IQR, 1.240-2.310) | n=243 | 0.9000 (IQR, 0.5800-1.650) | n=391 | <0.00010 |
| **Hemoglobin (g/dL)** | 12.15 (IQR, 9.925-13.60) | n=272 | 11.95 (IQR, 10.20-13.80) | n=486 | 0.57 |
| **Platelets (K/uL)** | 222 (IQR, 168.0-293.8) | n=272 | 220.0 (IQR, 152.0-287.0) | n=489 | 0.39 |
| **White blood cells (x10(3)/uL)** | 8.730 (IQR, 6.400-11.89) | n=272 | 8.805 (IQR, 6.358-13.60) | n=486 | 0.35 |
| **Absolute monocyte count (x10(3)/uL)** | 0.6700 (IQR, 0.4500-0.9575) | n=268 | 0.5800 (IQR, 0.3500-0.9250) | n=477 | 0.032 |
| **Absolute neutrophil count (x10(3)/uL)** | 6.220 (IQR, 3.998-9.225) | n=268 | 6.715 (IQR, 4.440-10.70 | n=478 | 0.016 |
| **Liver function** | | | | | |
| **Alanine transaminase (U/L)** | 21 (IQR, 15-34) | n=262 | 28 (IQR, 18-46.75) | n=484 | <0.00010 |
| **Aspartate aminotransferase (U/L)** | 29.0 (IQR, 21.0-52.0) | n=263 | 38.0 (IQR, 25.0-63.0) | n=484 | <0.00010 |
| **Total Protein (g/dL)** | 7.000 (IQR, 6.400-7.575) | n=264 | 6.800 (IQR, 6.300-7.300) | n=484 | 0.050 |
| **Albumin (g/dL)** | 3.900 (IQR, 3.425-4.300) | n=264 | 3.600 (IQR, 3.200-3.900) | n=484 | <0.00010 |
| **Bilirubin-D (mg/dL)** | 0.1000 (IQR, 0.1000-0.2000) | n=263 | 0.2000 (IQR, 0.1000-0.3000) | n=480 | 0.00030 |
| **Bilirubin-I (mg/dL)** | 0.3000 (IQR, 0.2000-0.5000) | n=243 | 0.3000 (IQR, 0.2000-0.5000) | n=454 | 0.71 |
| **Bilirubin-T (mg/dL)** | 0.5000 (IQR, 0.3000-0.8000) | n=264 | 0.5000 (IQR, 0.3000-0.8000) | n=482 | 0.060 |
| **Cardiac function** | | | | | |
| **Creatine kinase (U/L)** | 99.50 (IQR, 59.0-207.3) | n=254 | 126.5 (IQR, 66.25-30.5.5) | n=432 | 0.0074 |
| **Lactate dehydrogenase (U/L)** | 304.5 (IQR, 231.8-468.8) | n=258 | 405.0 (IQR, 303.5-598.5) | n=477 | <0.00010 |
| **Troponin T (ng/L)** | 22.00 (IQR, 10.00-52.50) | n=261 | 24.00 (IQR, 11.00-65.00) | n=434 | 0.33 |
| **Renal function** | | | | | |
| **Creatinine (mg/dL)** | 1.030(IQR, 0.7000-1.510) | n=271 | 1.060 (IQR, 0.7775-1.870) | n=486 | 0.042 |
| **Blood urea nitrogen (mg/dL)** | 18.00 (IQR, 12.00-32.00) | n=271 | 21.00 (IQR, 13.00-41.00) | n=486 | 0.0083 |
| **Inflammation** | | | | | |
| **C-reactive protein (mg/L)** | 19.1(IQR, 3.89-80.0) | n=263 | 109 (IQR, 32.5-196) | n=446 | <0.00010 |
| **Ferritin (mcg/L)** | 206 (IQR, 92.5-646) | n=252 | 592 (IQR, 322-1286) | n=441 | <0.00010 |
| **Fibrinogen (mg/dL)** | 438 (IQR, 341-581) | n=232 | 606 (IQR, 463-700) | n=391 | <0.00010 |
| **IL-6 (pg/mL)** | 29.2 (IQR, 8.70-103) | n=63 | 52.4 (IQR, 22.6-106) | n=281 | 0.014 |
| **Procalcitonin (ng/mL)** | 0.1300 (IQR, 0.07000-0.3625) | n=258 | 0.2700 (IQR, 0.1200-1.003) | n=434 | <0.00010 |
| **Coagulation** | | | | | |
| **D-Dimer (ug/mL)** | 1.78 (IQR, 0.750-3.160) | n=135 | 2.22 (IQR, 1.040-4.660) | n=294 | 0.0027 |
| **Antithrombin III (%)** | 109 (IQR, 94.0-122.0) | n=271 | 114.0 (IQR, 98.0-128.0) | n=488 | 0.0031 |
| **International normalized ratio (INR) test** | 1.200 (IQR, 1.100-1.300) | n=253 | 1.200 (IQR, 1.100-1.300) | n=461 | 0.59 |
| **Partial thromboplastin time (seconds)** | 31.50 (IQR, 28.68-35.40) | n=254 | 34.40 (IQR, 30.18-40.43) | n=458 | <0.00010 |
| **Prothrombin time (seconds)** | 14.60 (IQR, 13.70-15.80) | n=253 | 14.70 (IQR, 13.70-16.20) | n=462 | 0.47 |
| **Other parameters** | | | | | |
| **Glucose (mg/dL)** | 119 (IQR, 97.0-171.0) | n=271 | 132.5 (IQR, 109.0-192.3) | n=486 | <0.00010 |
| **Lactate(venous) (mmol/L)** | 1.600 (IQR, 1.200-2.500) | n=253 | 1.700 (IQR, 1.200-2.300) | n=403 | 0.74 |
